# Role of Flavohemoglobins in the Development and Aflatoxin Biosynthesis of *Aspergillus flavus*

**DOI:** 10.3390/jof10060437

**Published:** 2024-06-19

**Authors:** Xiaoling Zhou, Dongyue Chen, Min Yu, Yuan Jiao, Fang Tao

**Affiliations:** School of Life Sciences, Anhui Agricultural University, Hefei 230036, China; zhouxiaoling@stu.ahau.edu.cn (X.Z.); chendongyue@stu.ahau.edu.cn (D.C.); yumin@stu.ahau.edu.cn (M.Y.); jiaoyuan2023@ahau.edu.cn (Y.J.)

**Keywords:** *Aspergillus flavus*, flavohemoglobin, nitric oxide, fungal development, aflatoxin

## Abstract

*Aspergillus flavus* is notorious for contaminating food with its secondary metabolite—highly carcinogenic aflatoxins. In this study, we found that exogenous nitric oxide (NO) donor could influence aflatoxin production in *A. flavus*. Flavohemoglobins (FHbs) are vital functional units in maintaining nitric oxide (NO) homeostasis and are crucial for normal cell function. To investigate whether endogenous NO changes affect aflatoxin biosynthesis, two FHbs, FHbA and FHbB, were identified in this study. FHbA was confirmed as the main protein to maintain NO homeostasis, as its absence led to a significant increase in intracellular NO levels and heightened sensitivity to SNP stress. Dramatically, FHbA deletion retarded aflatoxin production. In addition, FHbA played important roles in mycelial growth, conidial germination, and sclerotial development, and response to oxidative stress and high-temperature stress. Although FHbB did not significantly impact the cellular NO level, it was also involved in sclerotial development, aflatoxin synthesis, and stress response. Our findings provide a new perspective for studying the regulatory mechanism of the development and secondary mechanism in *A. flavus*.

## 1. Introduction

*Aspergillus flavus* is a common saprophytic fungus. It is notorious for produce aflatoxins, a highly carcinogenic fungal toxin and classified as a Group 1 carcinogen to humans by the International Agency for Research (IARC) [1]. *A. flavus* primarily reproduces asexually through conidia. Under unfavorable environmental conditions, the strain forms a mycelial aggregate called sclerotium. Either sclerotia or conidia can survive for a long time and can germinate again for reproduction under favorable conditions. Once infected with crops or food, *A. flavus* can rapidly spread, affecting crop growth and food storage, thus severely impacting human health along the food chain [2,3]. Although the biosynthetic pathway of aflatoxin synthesis is known, the development and aflatoxin production of *A. flavus* are influenced by numerous factors, making the regulatory mechanisms highly complex. Currently, its prevention and control remain a global challenge.

Nitric oxide (NO) is a crucial signaling molecule first discovered in mammals, regulating various physiological processes like cell growth, apoptosis, neurotransmission, vasodilation, immunity, reproduction, and metabolism [4]. In bacteria, NO is involved in the formation and dispersion of biofilms and in the biosynthesis of secondary metabolites [5]. In plants, NO is involved in a myriad of bioprocesses such as the regulation of growth, development, photoperiod, flowering, plant immunity, and abiotic stress [6,7,8]. NO has also been found to play a role in the regulation of fungal growth, differentiation, and secondary metabolism [9,10,11]. For example, in *Neurospora crassa*, NO has been shown to inhibit conidiation and promote vegetative growth [12]. In *Aspergillus nidulans*, NO has been found to stimulate the production of sexual cleistothecia and decrease sterigmatocystin production [13]. In addition, NO has been implicated in the regulation of fungal virulence. For example, in the plant pathogenic fungus *Botrytis cinerea*, NO was required for full virulence on plants [14]. And in *Magnaporthe oryzae*, the removal of NO resulted in decreased pathogenicity [15]. NO also plays an important role in fungal response to abiotic and biotic stresses [16,17].

NO homeostasis is critical for normal cell function. NO has dual functions in eukaryotic organisms. NO-derived compounds produced inside the cells are either beneficial or deleterious to them. NO can serve as a precursor molecule for a series of reactive nitrogen species (RNS). Excessive accumulation of highly reactive RNS can lead to nitrosative stress, causing toxicity to cells [18]. To maintain the homeostasis of NO in the cell, different organisms have evolved NO detoxification mechanisms to combat RNS. Flavohemoglobin (FHb), or Nitric oxide dioxygenase (NOD), is a well-known enzyme involved in RNS response. FHb is a globin protein with a dual-domain structure. Its N-terminus contains a hemoglobin domain, while its C-terminus comprises an oxidoreductase domain with FAD and NAD(P)H binding sites [19]. Under aerobic conditions, FHb converts NO into a harmless nitrate, which can be stored (in plant vacuoles), secreted (in animals), or assimilated (in bacteria, fungi, and plants) [20,21]. Under anaerobic conditions, FHb has a reduction function for NO, converting it into non-toxic N_2_O [22,23]. In *Saccharomyces cerevisiae*, Yhb1 has been proved to be crucial for NO metabolism, safeguarding yeast from NO-related toxicity under both aerobic and anaerobic conditions [24,25]. In human pathogenic *Candida albicans*, CaYHB1 has been shown to be responsible for NO consumption and detoxification [26]. In *Aspergillus oryzae*, two FHbs, FHb1 and FHb2, were identified. Only deletion of FHb1 results in heightened cellular sensitivity to NO stress [27]. In *A. nidulans*, two FHbs, FHbA and FHbB, have been shown to be involved in the metabolism of NO to nitrate [28]. The knockout of *fhbA* in *A. nidulans* leads to an increase in cellular NO levels and a simultaneous decrease in the production of mycotoxin sterigmatocystin [13]. All the above suggest that FHb is crucial for NO homeostasis and plays important roles in fungal development and secondary metabolism. However, it is not yet reported whether the synthesis of aflatoxins is also influenced by NO.

In this study, to investigate the effect of NO on the aflatoxin biosynthesis, we first investigated the effects of exogenous NO on the aflatoxin biosynthesis. We then explored the effects of FHb gene deletion and overexpression on endogenous NO levels. Further, we assessed whether changes in endogenous NO levels affect aflatoxin production, as well as the mycelial growth, development, stress response, and pathogenicity of *A. flavus*.

## 2. Materials and Methods

### 2.1. Fungal Strains and Culture Conditions

The *A. flavus* NRRL 3357 was used as wild type (WT) strain throughout this work. WT and derivative transformants were routinely cultured at 28 °C on potato dextrose agar (PDA, BD Difco, Franklin Lakes, NJ, USA) or CDA (Czapek-Dox Broth (BD Difco, Franklin Lakes, NJ, USA) + 1.5% agar) medium to evaluate growth, colony characteristics, and conidiation. Wickerham medium was used to observe the formation of sclerotia [29]. YES medium (20 g/L yeast extract, 150 g/L sucrose, 15 g/L agar) was used for aflatoxin production analysis.

### 2.2. FHb Sequence Analysis

The FHb orthologs from different organisms were obtained from the NCBI database (http://www.ncbi.nlm.nih.gov/, accessed on 1 March 2022). The phylogenetic relationship was analyzed by MEGA-X [30]. Sequence alignments were performed using the Clustal W (version 2.0) [31]. Domain architecture was automatically provided by the SMART online software program (version 9) [32]. Further domain visualization of the proteins was completed by DOG2.0 (http://dog.biocuckoo.org, accessed on 1 June 2023).

### 2.3. FHb Deletion and Overexpression

The targeted gene deletion mutants were constructed following a previously established double-fluorescence knockout system [33]. The ~1.0 kb DNA fragments flanking upstream and downstream regions of the target gene *fhbA* were amplified with primers FHbA_5f/FHbA_5r and FHbA_3f/FHbA_3r. Flanking regions of target gene *fhbB* were amplified with primers FHbB_5f/FHbB_5r and FHbB_3f/FHbB_3r. The phleomycin-resistant gene and *RFP* gene fused cassette, Ble-RFP, was amplified from the pDHBG vector with the primers Pbr-f/Pbr-r (Table S1). The pUM-GFP vector containing the GFP expression cassette was linearized by *Bam*H I. All fragments were mixed and transformed into yeast strains FY834 to generate deletion construct pKO-FHbA and pKO-FHbB by the homologous recombination. These plasmids were purified and transformed to *Agrobacterium tumefaciens*, respectively. The fungal conidia were transformed using the *Agrobacterium tumefaciens*-mediated transformation (ATMT) method [34]. All phleomycin-resistant transformants were first screened by fluorescence. The transformants that only emitted red fluorescence were then screened for homogeneous nuclei (HMN) strains by negative screening double PCR [33] using primers FHbA-null-f/FHbA-null-r, FHbB-null-f/FHbB-null-r, and Ptub-f/Ptub-r.

The target gene overexpression strains were constructed following a previous site-specific integration system [35]. The foreign DNA integrated into the succinate dehydrogenase (SDH) gene site via a carboxin-resistant *sdh2^R^* allele (H249L) strategy. The left flank of *sdh2* harboring a point-mutated (H249L) site was generated with primer pairs P101/P102 and P103/P104. The right flank of *sdh2* was amplified using primers P105/P106. The *fhbA* and *fhbB* gene coding regions were amplified from WT genomic DNA using primer pairs OE::fhbA-f/OE::fhbA-r and OE::fhbB-f/OE::fhbB-r, respectively. The *gpdA* promoter and *trpC* terminator, used for overexpressing the target gene, were amplified from pDHBR plasmid [34]. All the above DNA fragments were cloned into the pUM vector [35] using a yeast recombination cloning approach to generate pUM::FHbA and pUM::FHbB vectors, respectively. The overexpression constructs were transformed into *A. flavus* WT through ATMT. The carboxin-resistant transformants were verified by PCR using primers P100-F/P100-R.

Primers used in this section are listed in Table S1.

### 2.4. qRT-PCR and Gene Expression Analysis

First, 10 μL of conidial suspension (1 × 10^6^ spores/mL) was seeded centrally onto the PDA plate and incubated at 28 °C for 3 d in darkness. Total RNA was isolated from the cultures using Trizol reagent (TaKaRa, Dalian, China) according to the manufacturer’s instructions. Then, 5 μg of total RNA was reversely transcribed into cDNA using a TransScript^®^ One-Step gDNA Removal and cDNA Synthesis SuperMix (Transgen, Co., Ltd., Beijing, China). Confirmation of deletions and overexpression of the *fhbA* and *fhbB* genes was made with primer pairs D-FHbA-f/D-FHbA-r and D-FHbB-f/D-FHbB-r, respectively. The *β-tubulin* gene amplified by primer pairs β-tubulin-F/β-tubulin-R was used as an endogenous control. Real-time quantitative RT-PCR was performed using the TB Green^®^ Premix Ex TaqTM II (TaKaRa Co., Ltd., Tokyo, Japan) following previously described procedures [33,36]. The *β-tubulin* gene was used as an endogenous control, with three biological replicates assessed for each sample. Relative expression levels were calculated using the comparative CT (2^−ΔΔCT^) method. Primers used in this section are listed in Table S1.

### 2.5. Phenotypic Characterization

To assess the fungal growth, 10 μL of conidial suspension (1 × 10^6^ spores/mL) was point inoculated onto fresh CDA and PDA media, followed by incubation at 30 °C for 5 d. The colony images were photographed at 5 d post inoculation (dpi). To evaluate sporulation, conidia of the tested strains were harvested from 5-day-old CDA and PDA agar plates using a solution of 0.01% Triton X-100 and counted using a hemocytometer. The conidial germination assay was conducted as follows: coverslips were placed in a Petri dish (90 mm). Then, 5 mL of YES medium was poured into the dish, before 1 μL of conidial suspension (1 × 10^6^ spores/mL) was inoculated onto each slip and cultured at 37 °C. Conidial germination was observed by microscopic examination of at least 1000 conidia per replicate for each time point. For sclerotia analysis, 10 μL of conidial suspension (1 × 10^6^ spores/mL) was seeded centrally onto the WKM plates and incubated in the dark at 30 °C for 10 d. The conidia were then washed off the plates with 75% alcohol and the remaining sclerotia were counted under a microscope. These experiments were repeated three times with three replicates each, and representative results from one experiment are shown.

### 2.6. Abiotic Stress Assay

For exogenous NO stress, sodium nitroprusside (SNP) was used for the NO donor [37]. Then, 10 µL of conidial suspension (1 × 10^6^ spores/mL) was inoculated onto YES plate supplementary SNP and cultured in darkness at 28 °C for 3 d. For oxidative stress, 10 µL of conidial suspension was inoculated onto CDA media supplementary H_2_O_2_ (3, 6, and 9 mM) and assessed after culturing at 28 °C for 4 d. For temperature stress, 10 µL of conidial suspension was inoculated onto CDA medium and cultured in darkness at 28 °C, 37 °C, and 42 °C for 4 d, respectively. The colony diameter of each strain was measured under all abiotic stress conditions. The growth inhibition rate for each strain under each stress condition was calculated by the method previously described [33,36]. All the experiments were repeated three times with triple replications.

### 2.7. Pathogenicity Assay

A laboratory kernel infection assay (KIA) was performed as previously described with slight modifications [33]. Undamaged maize kernels were sterilized with 75% ethanol and 1% NaClO for 5 min in turn. After being washed with distilled water, the kernels were immersed in a conidial suspension (2 × 10^6^/mL) and shaken at 70 r/min at 30 °C for 30 min. The kernels were then placed in 35 mm Petri dishes without a lid, and these small dishes were then placed in a large Petri dish (90 × 20 mm) with the embryo up and incubated at 30 °C for 10 d. The kernels immersed in distilled water served as the control and three replications were conducted for each test. Infection was designated as visible mycelia and conidia on the surface of the kernel. Spores were also harvested and counted with a hemacytometer.

### 2.8. Aflatoxin Extraction

According to our previously described method [33], aflatoxin extraction from mycelia was carried out with few modifications. Then, 10 µL of conidial suspension (1 × 10^6^ spore/mL) of each tested strain was inoculated onto the YES solid medium and cultured at 28 °C for 4 d. The fungal biomass was scraped from the plates and weighed, then extracted by incubation with 5 mL of methanol/water (7:3) at room temperature, with shaking at 200 rpm for 2 h. The supernatant was then collected by centrifugation at 3000× *g* for 10 min at room temperature and filtered through a syringe filter (0.22 µm, Alltech, Nicholasville, KY, USA). Aflatoxin extraction from infected maize kernels followed the methodology outlined in our previous studies [38].

To assess the effect of exogenous NO on the aflatoxins produced by the WT strain, 1 mL conidial suspension (7 × 10^8^/mL) of NRRL 3357 was inoculated in 150 mL of liquid YES medium and shaken at 100 r/min in darkness at 28 °C for 2 d. The cultures were then added to NO donor SNP and further incubated for 24 h and 48 h. After each duration, mycelia and liquid medium were harvested separately by filtering the culture through a miracloth. The medium was extracted with an equal volume of dichloromethane to isolate the aflatoxins. The mycelia were dried, weighed, and subjected to the same aflatoxin extraction procedure as in a solid culture.

### 2.9. UPLC-MS/MS Assay

An ACQUITY UPLC I-Class (Waters, Milford, MA, USA) and Xevo TQ-S triple quadrupole tandem mass spectrometer (Waters, Milford, MA, USA) were used to detect the AFB1. Separation of the AFB1 was carried out on an ACQUITY UPLC^®^BEH C 18 (2.1 mm × 50 mm, 1.7 µm, Waters, MA, USA). The elution solutions used were (A) 0.1% formic acid in water and (B) 0.1% formic acid in MeOH. The solutions were pumped at a flow rate of 0.3 mL/min, and A/B = 60:40 was applied. The column temperature was 50 °C. The injection volume of the samples was 2 µL. Waters MassLynx V4.2 SCN986 analysis software (Waters, MA, USA) was used to control the LC/MS/MS system and to acquire and process data. The mass spectrometer was operated in the positive electrospray ionization (ESI) mode with multiple reaction monitoring (MRM). The main MS parameters were optimized and finally set as follows: Capillary voltage, 3.5 KV; Cone voltages, 72 V; Desolvation temperature, 500 °C; Desolvation gas flow rate, 1000 L/h; Cone gas, 150 L/h; precursor ion (*m*/*z*), 313; and quantification ion (*m*/*z*), 285.

### 2.10. NO Detection Assay

Intracellular NO was detected using a Nitric Oxide Assay Kit (Beyotime, Shanghai, China), which is based on the classic Griess method. To quantify intracellular NO levels, fungal conidia were inoculated onto CDA medium overlaid with sterile cellophane sheets and cultured in darkness at 28 °C for 3 d. All mycelia were collected and weighed. The mycelia were subsequently disrupted using a JXFSTPRP-24L grinder (Jingxin, Shanghai, China) equipped with beads at a frequency of 60 Hz/s for 1 min, followed by the addition of 400 μL of ddH_2_O and centrifugation at 10,000× *g* for 15 min. The supernatant was boiled for 5 min and centrifuged at 12,000 rpm for 5 min. The resulting supernatant was then transferred to 96-well plates and the concentration of NO was measured according to the proceed of the kit (S0021S).

Intracellular NO was also visualized using DAF-FM DA (Beyotime, Shanghai, China). The conidia of all indicated strains were inoculated in CD liquid medium (Czapek-Dox Broth (BD Difco, Franklin Lakes, NJ, USA)) and shaken at 100 r/min in darkness at 28 °C for 20 h. The mycelia were collected and resuspended in 5 μΜ DAF-FM DA solution, followed by incubation at 37 °C for 20 min. The mycelia were then washed three times with PBS (pH 7.4). Fluorescence images were captured by laser confocal microscopy.

### 2.11. Statistical Analysis

All experimental results were reported as mean ± standard deviation (SD). Statistical analyses were performed with GraphPad Prism 8.0 software (GraphPad Software, San Diego, CA, USA) and a one-way ANOVA using Fisher’s LSD test. The significance level was set at *p* < 0.05.

## 3. Results

### 3.1. Exogenous NO Affects Aflatoxins Production in A. flavus

**Figure 1:**
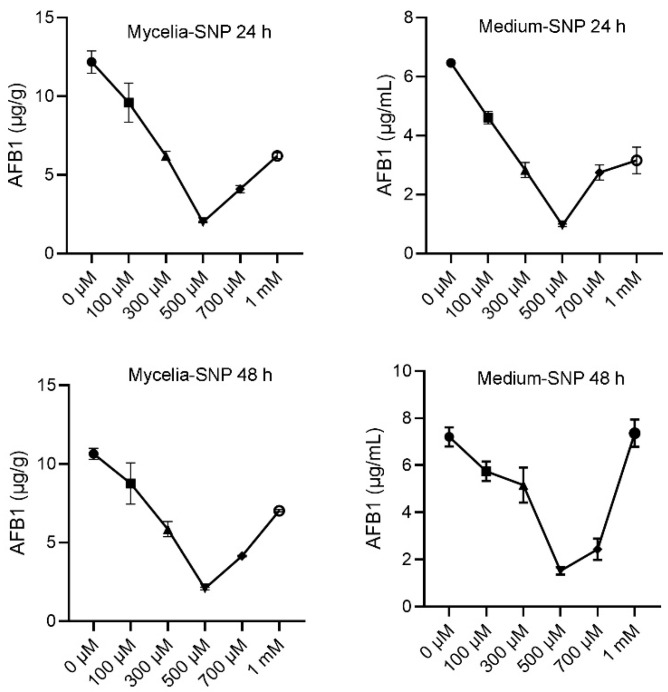
Effects of SNP on aflatoxin production of *A. flavus* WT. The results were presented as the mass of AFB1 per unit mass of fungal mycelium and the mass of AFB1 per milliliter liquid medium, respectively.

To investigate the effect of exogenous NO on the biosynthesis of aflatoxins in *A. flavus*, the appropriate concentration of SNP without affecting fungal growth should be determined. SNP was then used as an NO donor and added in YES liquid cultures, and the AFB1 was detected at 24 h and 48 h, respectively, after SNP addition. The results showed that concentrations of SNP below 1 mM did not impact the growth of *A. flavus* (Figure S1). However, exogenous NO had a significant effect on the production of AFB1. In the range of 0 μM to 500 μM SNP, as the concentration increased, both the mycelia and medium showed a significant decrease in the content of AFB1 in the mycelia, while in the range of 500 μM to 1 mM SNP, higher concentrations of NO resulted in a significant increase in AFB1 content in both the mycelia and culture medium (Figure 1). These findings suggest a threshold for the effect of NO on aflatoxin production in *A. flavus*. Within a certain threshold range, toxin production decreases as the concentration of NO increases.

### 3.2. Characterization of Flavohemoglobins in A. flavus

**Figure 2:**
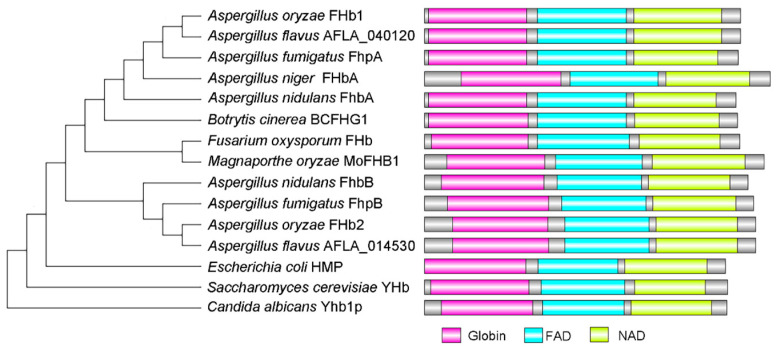
Phylogenetic analysis of flavohemoglobin orthologs from *A. flavus* and representative bacteria and fungi. AFLA_040120 and AFLA_014530 are the number of orthologs in *A. flavus.* The accession numbers of other sequences are XP_664773.1 *A. nidulans* FHbA, XP_661126.1 *A. nidulans* FHbB, CAF25490.1 *Aspergillus niger* FhbA, XP_001825874.1 *A. oryzae* FHb1, XP_001727230.1 *A. oryzae* FHb2, XP_746528.1 *Aspergillus fumigatus* FhpA, XP_747410.1 *A. fumigatus* FhpB, *B. cinerea* BCFHG1, XP_711046.1 *C. albicans* Yhb1p, CAP74387.1, BAA33011.1 *Fusarium oxysporum* FHb, XP_369046.1 *M. oryzae* MoFHB1, NP_011750.1 *S. cerevisiae* Yhb, and NP_417047.1 *E. coli* HMP. The phylogenetic tree of the 15 orthologs was constructed by MEGA-X. The domain was analyzed by InterProScan and drawn by DOG 2.0.

FHb proteins were identified by searching the *A. flavus* protein database based on the reported amino acid sequences of FHb proteins in fungi and bacteria. The results showed that, unlike bacteria and humans which typically have only one FHb, two orthologous proteins encoded by AFLA_040120 and AFLA_014530 were identified in *A. flavus* (Figure 2), similar to *A. oryzae* and *A. nidulans*. The AFLA_040120 and AFLA_014530 sequences are 1251 bp and 1311 bp long and encode a 416 amino acid protein and a 436 amino acid protein, respectively. By alignment with FHb1 and FHb2 in *A. oryzae* and FHbA and FHbB in *A. nidulans*, AFLA_040120 shares 99.04% amino acid sequence similarity with FHbA in *A. oryzae* and 67.31% similarity with FHbA in *A. nidulans*. AFLA_014530 exhibits 99.77% similarity with FHbB in *A. oryzae* and 72.50% similarity with FHbB in *A. nidulans* (Figure S2). Therefore, in this study, the genes AFLA_040120 and AFLA_014530 were named *fhbA* and *fhbB*, and the encoded proteins were named FHbA and FHbB, respectively. Protein domain analysis revealed that the predicted FHbA and FHbB of *A. flavus* contain three distinct domains: an N-terminal globin domain and C-terminal FAD and NAD binding domains (Figure 2), which is consistent with the first deciphered structure of the *Escherichia coli* HMP protein [19].

### 3.3. Construction of fhbA and fhbB Deletion and Overexpression Mutants

**Figure 3:**
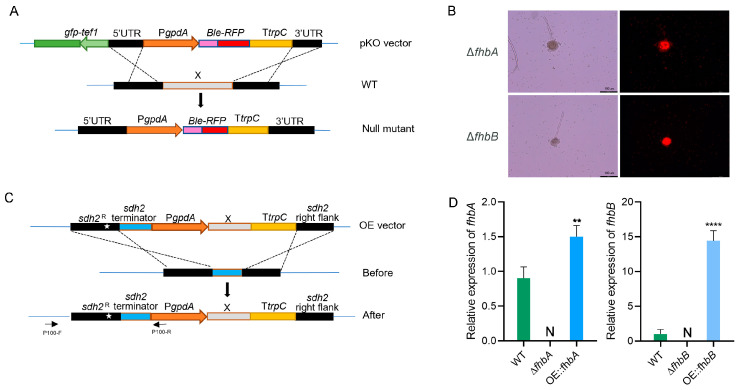
Knockout and overexpression of *fhbA* and *fhbB*. (**A**) Gene knockout strategy in *A. flavus*. X: *fhbA* and *fhbB*, respectively; WT: the WT strain. (**B**) The putative knockout null mutant with red fluorescence. (**C**) Gene overexpression strategy in *A. flavus*. The white star stands for the *sdh2* mutation site. (**D**) The expression levels of *fhbA* and *fhbB* in the WT, the gene-deleted mutant Δ*fhbA* and Δ*fhbB*, and gene-overexpressed strain OE::*fhbA* and OE::*fhbB*. “N” stands for “Not Detected”. ** *p* < 0.01, **** *p* < 0.0001.

The *fhbA* and *fhbB* gene knockout strains were constructed using homologous recombination. The principle of constructing the gene knockout strain is shown in Figure 3A. The T-DNA region contained a Ble-RFP cassette, and the 5′-up sequence and 3′-down sequence of the target gene (*fhbA* or *fhbB*) were located on both sides of this cassette. The T-DNA region also contained a GFP gene expression cassette which was used to exclude ectopic transformants. During homologous recombination, the Ble-RFP cassette replaced the target gene. Only those with red fluorescence are putative null mutants [33]. As shown in Figure 3B, the Δ*fhbA* and Δ*fhbB* strains only exhibited red fluorescence, indicating that they are putative null mutants. To screen for the null mutant with homogeneous nuclei (HMN), double-PCR was performed to detect the target gene and *β-tubulin*. Only one band corresponding to *β-tubulin* could be amplified from the HMN strain (Figure S3A), which suggests that the *fhbA* and *fhbB* genes were knocked out, respectively.

The *fhbA* and *fhbB* gene overexpression strains were constructed using a site-specific integration system. The construction strategy is shown in Figure 3C. The T-DNA region contained the *PgpdA* promoter, the *TrpC* terminator, and the sequence of the target gene (*fhbA* or *fhbB*). It also included the *sdh2* left flanking fragment (containing the *sdh2* mutation site) and the *sdh2* right flanking fragment, which were used for homologous recombination after transformation to insert the exogenous fragment into the *sdh2* locus. Carboxin resistance selection was then performed, and the resulting transformants were screened and verified by positive PCR with primer pairs P100-F/P100-R and *β-tubulin* as controls. The results showed that a band of approximately 1.9 kb was amplified from the transformant but not from the WT (Figure S3B), indicating that the target genes have been inserted into the specific site.

The relative expression levels of genes *fhbA* and *fhbB* were determined using qRT-PCR in the knockout and overexpression strains. In the Δ*fhbA* and Δ*fhbB* mutants, no expression of the target genes was detected. In the OE::*fhbA* strain, the expression level of *fhbA* was 1.5-fold higher than that in the WT strain, while in the OE::*fhbB* strain, the expression level of *fhbB* was 14-fold higher than that in the WT strain (Figure 3). The results also further confirmed the successful knockout and overexpression of the target genes *fhbA* and *fhbB*.

### 3.4. fhbA Plays a Key Role in Sustaining NO Homeostasis in A. flavus

**Figure 4:**
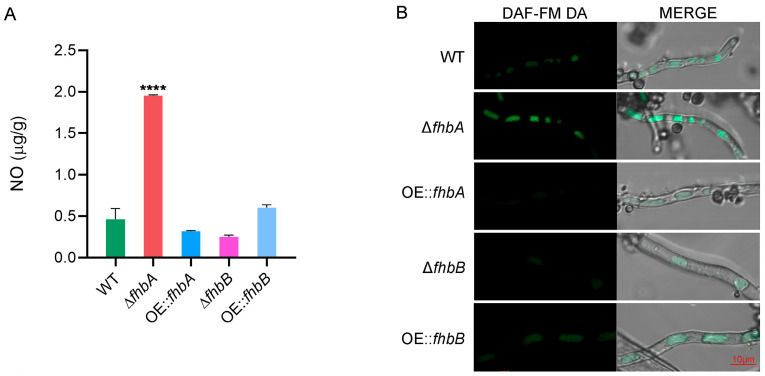
Effect of *fhbA* and *fhbB* on intracellular NO in *A. flavus*. (**A**) NO level tested by the Griess method. The level of NO was presented as the mass of NO per unit mass of fungal mycelium. **** *p* < 0.0001. (**B**) *A. flavus* strains were stained with DAF-FM DA and imaged by laser confocal microscopy.

**Figure 5:**
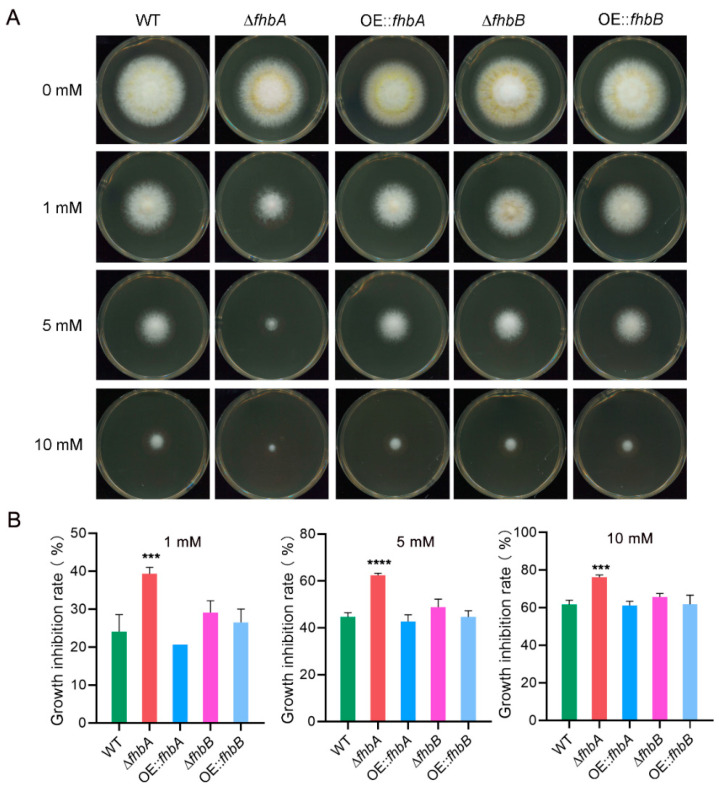
Effects of SNP stress on growth of *A. flavus* strains. (**A**) Colonies of the indicated strains subjected to different concentrations of SNP. All strains were inoculated onto YES supplemented with SNP at 28 °C in darkness for 3 d. (**B**) Growth inhibition rate of mutants under SNP stress. The rate of inhibition of mycelial growth was calculated by measuring the diameter of fungal colonies and normalized to the growth of control, respectively. *** *p* < 0.001, **** *p* < 0.0001.

FHb plays a crucial role in sustaining NO homeostasis within the cell by clearing excess NO [20]. To ascertain whether FHb mediated the same role in *A. flavus*, we first compared the endogenous NO levels in the WT and the *fhbA* and *fhbB* deletion and overexpression strains. The results showed that the NO content increased by approximately 4.2-fold in Δ*fhbA* compared with the WT (Figure 4A), whereas other mutants showed no significant difference to the WT. The intracellular NO was also visualized using DAF-FM DA, the NO-sensitive fluorescent probes. The fluorescence within the hyphae of the Δ*fhbA* mutant strain was stronger than that of the WT and other mutant strains (Figure 4B). We further treated the WT and the four mutant strains with SNP. The WT exhibited significant growth inhibition when treated with SNP concentrations above 1 mM and could not grow above 20 mM SNP (Figure S4). Only Δ*fhbA* was more sensitive to SNP, with a higher growth inhibition rate than the WT at concentrations of 1 mM–10 mM (Figure 5). Our results indicate that *fhbA* plays an important role in eliminating excess NO in *A. flavus*.

### 3.5. Different Role of fhbA and fhbB in the Biosynthesis of AFB1

**Figure 6:**
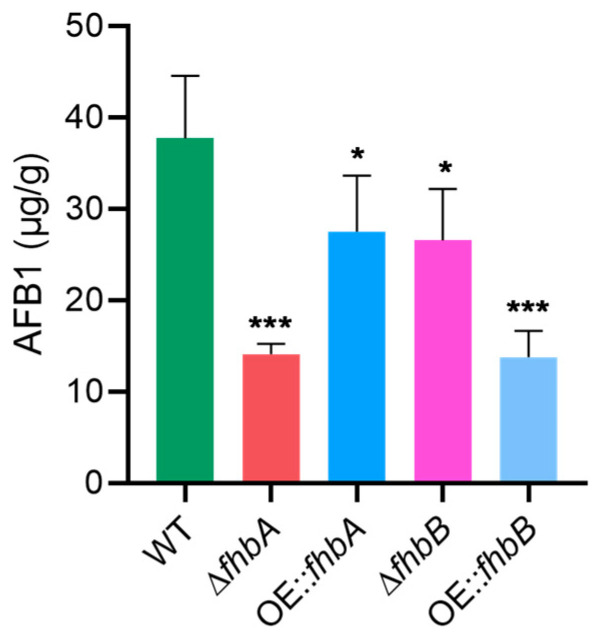
Effect of *fhbA* and *fhbB* on AFB1 production. The results were presented as the mass of AFB1 per unit mass of fungal mycelium. * *p* < 0.05, *** *p* < 0.001.

To assess the impact of *fhbA* and *fhbB* on AFB1 production in *A. flavus*, LC-MS was performed to measure the content of AFB1 in various strains cultured onto YES plate. The results revealed that either deletion or overexpression of *fhbA* and *fhbB*, respectively, significantly reduced AFB1 production. In particular, deletion of *fhbA* resulted in a 63% decrease in AFB1 levels compared to the WT, whereas gene overexpression resulted in a 27% decrease. Conversely, deletion of *fhbB* resulted in a 30% reduction in AFB1 levels compared to the WT, whereas overexpression of the gene resulted in a 63% decrease (Figure 6). These results indicate that both *fhbA* and *fhbB* are involved in the regulation of aflatoxin synthesis, but they might exert different effects on the regulation of toxin biosynthesis.

### 3.6. fhbA Affects Mycelial Growth

**Figure 7:**
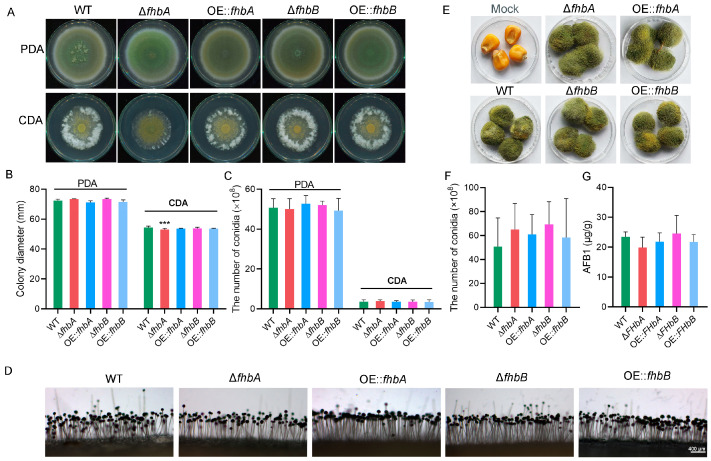
Effect of *fhbA* and *fhbB* on growth of *A. flavus*. (**A**) Phenotype of mycelia grown on PDA and CDA medium. All tested strains were inoculated onto PDA and CDA at 30 °C for 5 d and then photographed. (**B**) Mycelia growth rate analysis. Colony diameters of the tested strains on PDA and CDA media were measured and analyzed. *** *p* < 0.001. (**C**) Conidial production analysis on PDA and CDA plate. (**D**) Conidiophores of the mutant strains. (**E**) Virulence assay of mutants on maize kernels. (**F**) Conidial production on kernels. (**G**) AFB1 production in maize kernels infected with *A. flavus* strains. The results were presented as the mass of AFB1 per unit mass of infected kernels.

To investigate the role of *fhbA* and *fhbB* on the mycelium growth and conidiogenesis in *A. flavus*, the mycelial growth of WT, the Δ*fhbA* and Δ*fhbB* strains, and OE::*fhbA* and OE::*fhbB* strains were compared on PDA and CDA medium. Only the growth of Δ*fhbA* strain exhibited a significant difference compared to the WT (Figure 7A,B). Meanwhile, the Δ*fhbA* developed a lighter color of colonies on PDA medium and fewer aerial hyphae on CDA medium compared to the WT (Figure 7A). The conidiation of all strains were analyzed and showed that there were no significant differences between the mutants and the WT (Figure 7C). Neither deletion nor overexpression of *fhbA* and *fhbB* had any effect on the conidiophore development (Figure 7D).

To further understand the role of *fhbA* and *fhbB* in pathogenic development, conidial suspension of all tested strains was inoculated with maize kernels. The results indicated that there were no significant differences in the extent of grain surface coverage by hyphae and spores between the mutant strains and the WT strain (Figure 7E). Similarly, there were no significant differences in spore and AFB1 production levels among the different strains during maize infection (Figure 7F,G).

### 3.7. fhbA Promotes Conidial Germination

**Figure 8:**
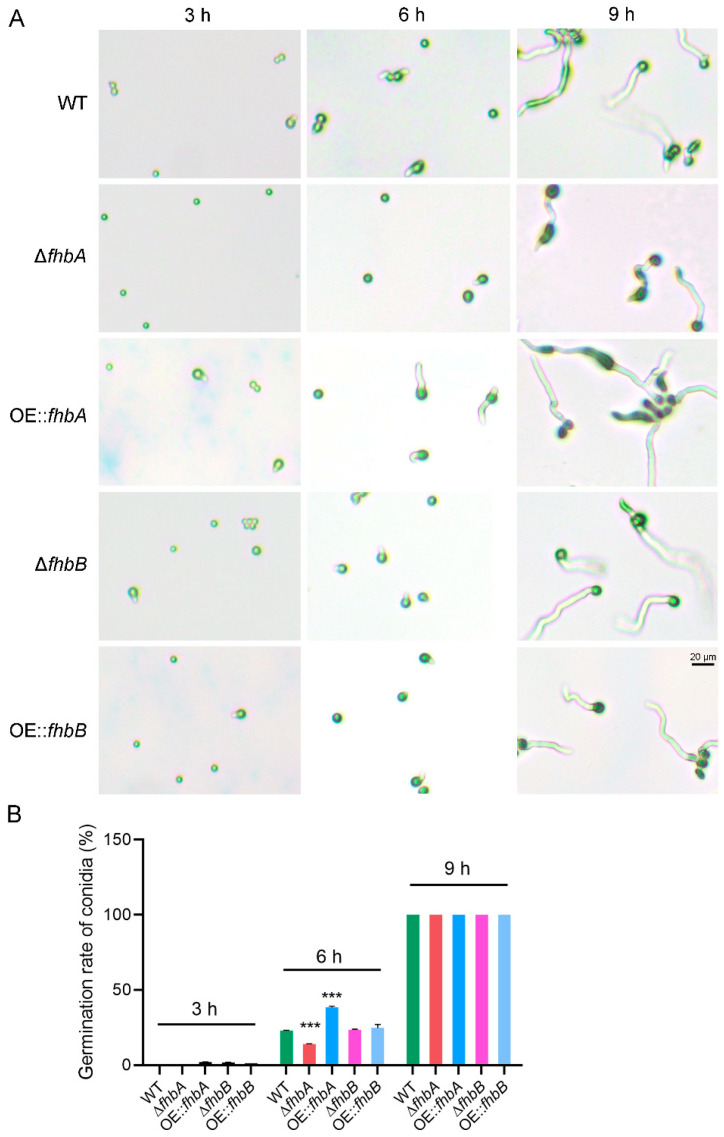
Effect of *fhbA* and *fhbB* on conidial germination. (**A**) The morphology of germinated conidia from *fhbA* and *fhbB* deletion mutant and overexpression strains. (**B**) Statistical analysis of conidial germination. Germinated conidia were counted at each indicated time under a light microscope. *** *p* < 0.001.

To ascertain the role of FHbs in *A. flavus* conidia germination, all mutant strains and the WT strain were cultured on coverslips. The results showed that at 3 h post inoculation (hpi), the conidia from all test strains except for the Δ*fhbA* strain had just initiated germination, with no significant differences observed. At 6 hpi, the germination rate of WT conidia was 23.2%. In comparison, conidial germination significantly decreased to 14.2% in Δ*fhbA* and increased to 38.9% in OE::*fhbA*. However, Δ*fhbB* and OE::*fhbB* showed no difference in conidial germination compared to the WT. At 9 h, 100% conidia of all tested strains had germinated. However, Δ*fhbA* exhibited shorter hyphae and OE::*fhbA* showed relatively longer hyphae compared to the WT (Figure 8). These results suggest that *fhbA* may play a role in conidial germination in *A. flavus*.

### 3.8. fhbs Involved in Sclerotial Development

**Figure 9:**
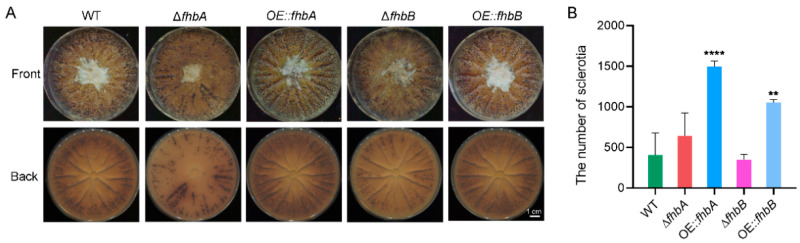
Effect of *fhbA* and *fhbB* on sclerotial development. (**A**) Colonies of the indicated strains with sclerotia on WKM media. (**B**) The number of sclerotia per plate. ** *p* < 0.01, **** *p* < 0.0001.

Sclerotia, the robust and compact structures formed by *A. flavus* under unfavorable environmental conditions, have been recognized as vital reproductive bodies. To identify the effect of deletion and overexpression of FHb on sclerotia development in *A. flavus*, conidia of each strain were inoculated onto WKM medium and subjected to a 10-day dark cultivation period. Surprisingly, the deletion of *fhbA* and *fhbB* did not significantly affect the production of sclerotia. However, when *fhbA* and *fhbB* were overexpressed, a substantial increase in sclerotium production was observed compared to the WT strain, with 2.7-fold increase in OE::*fhbA* and 1.9-fold increase in OE::*fhbB*, respectively (Figure 9).

### 3.9. fhbA and fhbB Response to Oxidative Stress

**Figure 10:**
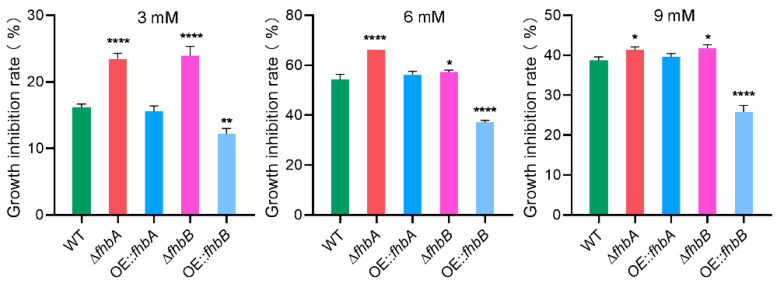
Effects of different concentrations of H_2_O_2_ on growth of *A. flavus* strains. Growth inhibition rate of the indicated strains under H_2_O_2_ stress. * *p* < 0.05, ** *p* < 0.01, **** *p* < 0.0001.

The ability to withstand oxidative stress is crucial for maintaining normal cellular functions in fungi, and it is closely associated with their secondary metabolism. To investigate the roles of FHb in the response of *A. flavus* to oxidative stress, the WT and FHb deletion and overexpression strains were subjected to H_2_O_2_-induced stress. The results showed that under all tested concentration conditions (3 mM, 6 mM, and 9 mM), deletion of *fhbA* and *fhbB* decreased the resistance of *A. flavus* to oxidative stress, while overexpression of *fhbB* enhanced its resistance to oxidative stress (Figure 10 and Figure S5). These findings suggest that *fhbA* and *fhbB* are both involved in the defense against oxidative stress.

### 3.10. Both fhbA and fhbB Response to High Temperatures

**Figure 11:**
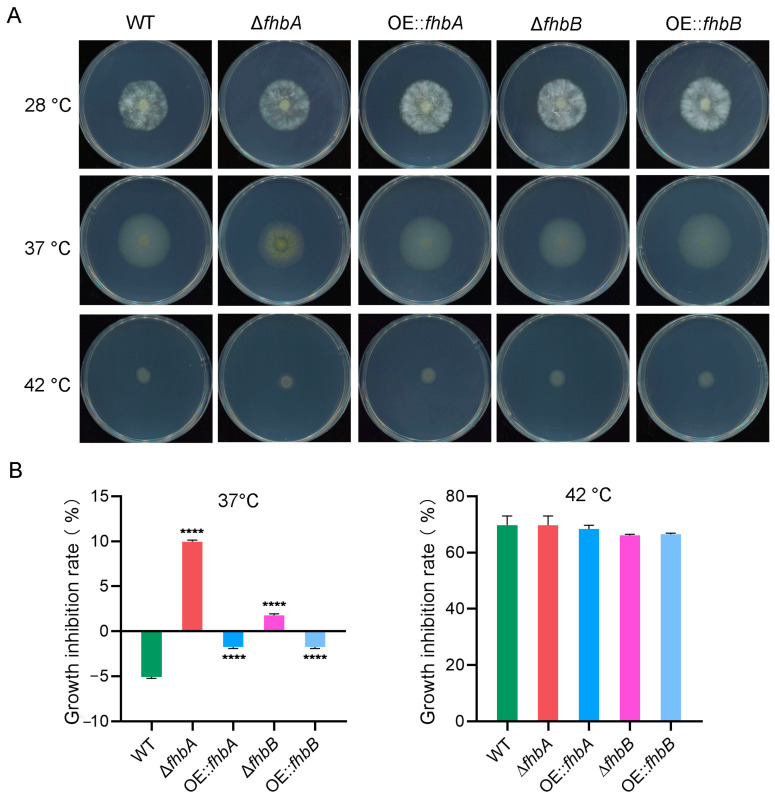
Temperature stress assay in *A. flavus*. (**A**) Colonies of the indicated strains cultured at higher temperatures (37 °C and 42 °C). The strains cultured at 28 °C were used as control. (**B**) Growth inhibition rate of the indicated strains at higher temperatures. **** *p* < 0.0001.

To further explore the role of FHb in response to the high temperatures in *A. flavus*, the mycelial growth of the mutant strains and WT at high temperatures (37 °C and 42 °C, respectively) were compared with that at 28 °C. The colony growth of Δ*fhbA* and Δ*fhbB* at 37 °C were all dramatically inhibited compared with the increased growth of WT. Meanwhile, 37 °C also promoted the growth of OE::*fhbA* and OE::*fhbB*, but there was a significant difference compared to the WT (Figure 11), while at 42 °C, the growth of all mutants was inhibited and showed no difference compared to the WT. The results indicate that *fhbA* and *fhbB* play a significant role in combating higher temperature stress.

## 4. Discussion

FHb, known as flavohemoglobin, was first discovered in *E. coli* (named HMP) and was hailed as a defense mechanism against the toxic effects of bacterial-produced nitric oxide (NO) [39]. Subsequent studies confirmed FHb in eukaryotic organisms, including yeast, filamentous fungi, and plants. The number of FHb orthologs varies across species. Only one FHb ortholog was founded in *S. cerevisiae* [24], *Cryptococcus neoformans* [40], and *B. cinerea* [41]. In the *C. albicans*, three FHb orthologs were found, but only CaYHb1 demonstrates NOD activity and assumes responsibility for NO clearance [26]. In *A. oryzae*, there are two FHb orthologs, FHb1 and FHb2, both of which exhibit NOD activity [42]. Only the *fhb1* gene responded to external nitric oxide (NO) stress at the transcriptional level. Disrupting *fhb1* increased cell hypersensitivity to NO stress [43]. In this study, two FHb orthologs, FHbA and FHbB, were identified in the *A. flavus*, both of which were more than 99% homologous to FHb1 and FHb2 in *A. oryzae*.

We further confirmed that FHbA plays a key role in endogenous NO elimination, as deletion of the *fhbA* gene leads to significantly elevated NO levels and increased sensitivity to SNP stress. However, overexpression of *fhbA* did not decrease the NO level. We speculate that cells require a certain concentration of NO to maintain normal cellular functions. Although overexpression of the *fhbA* gene may lead to the clearance of NO within cells, NO continues to be synthesized to maintain NO homeostasis. This precisely indicates that FHbA is the primary enzyme responsible for NO clearance, because the increase in NO caused by the deletion of the *fhbA* gene is not alleviated by other pathways. Furthermore, whether the *fhbB* gene was deleted or overexpressed, its effect on intracellular NO levels was not significant. It has been reported that in *A. oryzae*, the N-terminus of FHbB possesses a mitochondrial targeting signal, and FHbB is localized in mitochondria, potentially participating in mitochondrial NO stress responses [42,43]. In this study, the homology between the FHb proteins of *A. flavus* and those in *A. oryzae* exceeds 99%; the N-terminus of *A. flavus* FHbB also carries a potential mitochondrial targeting signal (Figure S2). Therefore, it is inferred that FHbB in *A. flavus* may also engage in mitochondrial NO stress responses, with minimal impact on cellular NO levels as a whole. And it is a challenge to detect the influence of FHbB on cellular NO levels using existing detection methods.

FHB regulates the level of intracellular NO and participates in fungal secondary metabolism. In this study, we found that there was a threshold of the exogenous NO level which affected AFB1 production in *A. flavus*. NO concentration below a certain threshold (<500 μM SNP) could reduce AFB1 production, but above this threshold, AFB1 production in *A. flavus* increased. Moreover, the intracellular NO level in the Δ*fhbA* strain significantly increased, while its AFB1 production decreased significantly, suggesting that within a certain threshold range, the NO level was negatively correlated with AFB1 production. In *A. nidulans*, deletion of *fhbA* also increases intracellular NO levels and reduces sterigmatocystin production [13]. Curiously, the NO level in other mutant strains was comparable to those in the WT, but the AFB1 production remarkably reduced, especially the *OE::fhbB*. We speculate that FHbs might regulate aflatoxin biosynthesis not only by a NO signal but also through other unknown pathways. Additionally, although the AFB1 production of various mutant strains on the YES agar medium was significantly lower than that of the WT, there was no significant difference in toxin production on infected maize kernels compared to the WT. This may be due to some unknown interactions between kernels and fungi.

FHb is involved in the regulation of growth and development in some fungi. In *A. oryzae*, deletion of the *fhb1* gene negatively affects mycelial growth [43]. Moreover, in a *pclA*-disrupted strain with a hyperbranching growth phenotype, the transcript levels of the *fhbA* gene were 2–5 times higher compared to the WT, which indicate a functional correlation between the *fhbA* gene and mycelial growth in *A. oryzae* [44]. In *M. oryzae*, although the expression of the *MoFHB1* (*fhbA* ortholog) was developmentally regulated during conidial germination and appressorium development, disruption of *MoFHB1* did not change vegetative growth, conidiation, and virulence [45]. In this study, the deletion and overexpression mutants of *fhbA* and *fhbB* had no impact on conidiogenesis and pathogenicity. However, Δ*fhbA* showed slower vegetative growth on CDA medium compared to the WT. Further, we found that deletion of *fhbA* delayed conidial germination, whereas overexpression of *fhbA* promoted spore germination. This suggests that *fhbA* may play a positive regulatory role in the conidial germination stage. In addition, deletion of the *fhbA* or *fhbB* genes in *A. flavus* did not affect sclerotial development, while overexpression of these genes increased the number of sclerotia. In contrast, in *A. nidulans*, deletion of *fhbA* increased the number of Hülle cells (precursors of cleistothecia) [13]. Hence, we speculate that FHb might have different regulatory mechanisms in growth and development among different fungal species. In addition, Yang et al. recently reported that the *fhb1* deletion mutant produced much more sclerotia than the *A. flavus* wild type [46]. This difference could potentially be attributed to variations in the genetic background of the strains utilized or in the conditions for sclerotial cultivation.

Apart from its role in NO metabolism and detoxification, FHb has been proposed to participate in oxidative stress. In *S. cerevisiae*, treatment with antimycin A or thiol oxidants, or lacking superoxide dismutase, resulted in the accumulation of reactive oxygen species and enhanced expression of YHb1. Additionally, YHb1 deficiency made yeast cells more sensitive to oxidative conditions, indicating a role for YHb1 in oxidative stress response [25]. In *A. oryzae*, *fhbA* and *fhbB* deletion strains exhibited higher resistance to H_2_O_2_ compared to the WT, while the *fhbB* overexpression strain showed hypersensitivity to H_2_O_2_, indicating a detrimental role in defending against H_2_O_2_ [27]. However, the results of this study revealed that deletion strains Δ*fhbA* and Δ*fhbB* displayed decreased resistance to H_2_O_2_, while the overexpression strain of *fhb*B exhibited heightened resistance compared to the WT, contrary to the findings observed in *A. oryzae*.

In addition, previous studies have shown that an increase in endogenous NO levels leads to increased heat tolerance in *S. cerevisiae* [47]. In contrast, deletion of *fhbA* in *A. flavus* resulted in dramatically elevated NO levels, which inhibited vegetable growth at 37 °C compared to 28 °C. We speculated that there might be two reasons for this: (1) NO has a dual effect on the regulation of some bioprocesses based on dose–effect. High concentrations of NO may exert opposite effects in certain biological processes compared to low concentrations. (2) FHb may influence strain response to heat stress not only by regulating NO levels, but also through other unidentified biological pathways. Regardless, FHbs play crucial roles in fungal tolerance to high-temperature environmental stress.

## 5. Conclusions

In this study, we confirmed that exogenous NO could affect AFB1 synthesis in *A. flavus*. Two flavohemoglobins, FHbA and FHbB, were identified in *A. flavus*. FHbA played a key role in sustaining NO homeostasis and participated in mycelial growth, conidial germination, sclerotial development, AFB1 biosynthesis, and response to oxidative stress and temperature stress. FHbB participated in sclerotial development, toxin synthesis, and response to stress. This research for the first time clarified the important role of FHbs in the development and aflatoxins synthesis in *A. flavus*, providing a new direction for the study of the regulatory mechanism of *A. flavus* growth and development.

## Data Availability

Data are contained within this article or in Supplementary Materials.

## Supplementary Materials

The following supporting information can be downloaded at https://www.mdpi.com/article/10.3390/jof10060437/s1, Figure S1: Effects of SNP on the growth of *A. flavus* WT strain; Figure S2: Primary sequence alignment of FHbA and FHbB from *A. flavus*, *A. oryzae*, and *A. nidulans*; Figure S3: The genes deletion mutant and overexpression strain were validated by PCR; Figure S4: Effects of SNP on the growth of WT strain; Figure S5: Colonies of the indicated strains subjected to H_2_O_2_; Table S1: Primers used in this study.

## References

[1] IARC Working Group on the Evaluation of Carcinogenic Risks to Humans, International Agency for Research on Cancer, World Health Organization (2002). Some Traditional Herbal Medicines, Some Mycotoxins, Naphthalene and Styrene (No.82).

[2] Kumar P., Mahato D.K., Kamle M., Mohanta T.K., Kang S.G. (2016). Aflatoxins: A global concern for food safety, uuman health and their management. Front. Microbiol..

[3] Mahato D.K., Lee K.E., Kamle M., Devi S., Dewangan K.N., Kumar P., Kang S.G. (2019). Aflatoxins in food and feed: An overview on prevalence, detection and control strategies. Front. Microbiol..

[4] Lundberg J.O., Weitzberg E. (2022). Nitric oxide signaling in health and disease. Cell.

[5] Chen J., Liu L., Wang W., Gao H. (2022). Nitric oxide, nitric oxide formers and their physiological impacts in bacteria. Int. J. Mol. Sci..

[6] Brouquisse R. (2019). Multifaceted roles of nitric oxide in plants. J. Exp. Bot..

[7] He Y., Tang R.H., Hao Y., Stevens R.D., Cook C.W., Ahn S.M., Jing L., Yang Z., Chen L., Guo F. (2004). Nitric oxide represses the *Arabidopsis* floral transition. Science.

[8] Yu M., Lamattina L., Spoel S.H., Loake G.J. (2014). Nitric oxide function in plant biology: A redox cue in deconvolution. New Phytol..

[9] Cánovas D., Marcos J.F., Marcos A.T., Strauss J. (2016). Nitric oxide in fungi: Is there NO light at the end of the tunnel?. Curr. Genet..

[10] Astuti R.I., Nasuno R., Takagi H. (2018). Nitric oxide signalling in yeast. Nitric Oxide and Other Small Signalling Molecules.

[11] Zhao Y., Lim J., Xu J., Yu J.H., Zheng W. (2020). Nitric oxide as a developmental and metabolic signal in filamentous fungi. Mol. Microbiol..

[12] Pengkit A., Jeon S.S., Son S.J., Shin J.H., Baik K.Y., Choi E.H., Park G. (2016). Identification and functional analysis of endogenous nitric oxide in a filamentous fungus. Sci. Rep..

[13] Baidya S., Cary J.W., Grayburn W.S., Calvo A.M. (2011). Role of nitric oxide and flavohemoglobin homolog genes in *Aspergillus nidulans* sexual development and mycotoxin production. Appl. Environ. Microbiol..

[14] Turrion-Gomez J.L., Benito E.P. (2011). Flux of nitric oxide between the necrotrophic pathogen *Botrytis cinerea* and the host plant. Mol. Plant Pathol..

[15] Samalova M., Johnson J., Illes M., Kelly S., Fricker M., Gurr S. (2013). Nitric oxide generated by the rice blast fungus *Magnaporthe oryzae* drives plant infection. New Phytol..

[16] Astuti R.I., Watanabe D., Takagi H. (2016). Nitric oxide signaling and its role in oxidative stress response in *Schizosaccharomyces pombe*. Nitric Oxide.

[17] Luo L., Zhang S., Wu J., Sun X., Ma A. (2021). Heat stress in macrofungi: Effects and response mechanisms. Appl. Microbiol. Biotechnol..

[18] Ridnour L.A., Thomas D.D., Mancardi D., Espey M.G., Miranda K.M., Paolocci N., Feelisch M., Fukuto J., Wink D.A. (2004). The chemistry of nitrosative stress induced by nitric oxide and reactive nitrogen oxide species. Putting perspective on stressful biological situations. Biol. Chem..

[19] Ilari A., Boffi A. (2008). Structural studies on flavohemoglobins. Methods Enzymol..

[20] Forrester M.T., Foster M.W. (2012). Protection from nitrosative stress: A central role for microbial flavohemoglobin. Free Radic. Biol. Med..

[21] Gardner P.R., Gardner A.M., Martin L.A., Dou Y., Li T., Olson J.S., Zhu H., Riggs A.F. (2000). Nitric-oxide dioxygenase activity and function of flavohemoglobins. sensitivity to nitric oxide and carbon monoxide inhibition. J. Biol. Chem..

[22] Liu L., Zeng M., Hausladen A., Heitman J., Stamler J.S. (2000). Protection from nitrosative stress by yeast flavohemoglobin. Proc. Natl. Acad. Sci. USA.

[23] Nakano M.M. (2006). Essential role of flavohemoglobin in long-term anaerobic survival of *Bacillus subtilis*. J. Bacteriol..

[24] Cassanova N., O’Brien K.M., Stahl B.T., McClure T., Poyton R.O. (2005). Yeast flavohemoglobin, a nitric oxide oxidoreductase, is located in both the cytosol and the mitochondrial matrix: Effects of respiration, anoxia, and the mitochondrial genome on its intracellular level and distribution. J. Biol. Chem..

[25] Zhao X.J., Raitt D., Burke P.V., Clewell A.S., Kwast K.E., Poyton R.O. (1996). Function and expression of flavohemoglobin in *Saccharomyces cerevisiae*. Evidence for a role in the oxidative stress response. J. Biol. Chem..

[26] Ullmann B.D., Myers H., Chiranand W., Lazzell A.L., Zhao Q., Vega L.A., Lopez-Ribot J.L., Gardner P.R., Gustin M.C. (2004). Inducible defense mechanism against nitric oxide in *Candida albicans*. Eukaryot. Cell.

[27] Zhou S., Fushinobu S., Kim S.W., Nakanishi Y., Wakagi T., Shoun H. (2010). *Aspergillus oryzae* flavohemoglobins promote oxidative damage by hydrogen peroxide. Biochem. Biophys. Res. Commun..

[28] Schinko T., Berger H., Lee W., Gallmetzer A., Pirker K., Pachlinger R., Buchner I., Reichenauer T., Güldener U., Strauss J. (2010). Transcriptome analysis of nitrate assimilation in *Aspergillus nidulans* reveals connections to nitric oxide metabolism. Mol. Microbiol..

[29] Chang P.K., Scharfenstein L.L., Li R.W., Arroyo-Manzanares N., De Saeger S., Diana Di Mavungu J. (2017). *Aspergillus flavus* aswA, a gene homolog of *Aspergillus nidulans* oefC, regulates sclerotial development and biosynthesis of sclerotium-associated secondary metabolites. Fungal Genet. Biol..

[30] Kumar S., Stecher G., Li M., Knyaz C., Tamura K. (2018). MEGA X: Molecular evolutionary genetics analysis across computing platforms. Mol. Biol. Evol..

[31] Larkin M.A., Blackshields G., Brown N.P., Chenna R., McGettigan P.A., McWilliam H., Valentin F., Wallace I.M., Wilm A., Lopez R. (2007). Clustal W and Clustal X version 2.0. Bioinformatics.

[32] Letunic I., Khedkar S., Bork P. (2021). SMART: Recent updates, new developments and status in 2020. Nucleic Acids Res..

[33] Zhao Q., Pei H., Zhou X., Zhao K., Yu M., Han G., Fan J., Tao F. (2022). Systematic characterization of bZIP transcription factors required for development and aflatoxin generation by high-throughput gene knockout in *Aspergillus flavus*. J. Fungi.

[34] Han G., Shao Q., Li C., Zhao K., Jiang L., Fan J., Jiang H., Tao F. (2018). An efficient *Agrobacterium*-mediated transformation method for aflatoxin generation fungus *Aspergillus flavus*. J. Microbiol..

[35] Tao F., Zhao K., Zhao Q., Xiang F., Han G. (2020). A novel site-specific integration system for genetic modification of *Aspergillus flavus*. G3-Genes Genomes Genet..

[36] Yu M., Zhou X., Chen D., Jiao Y., Han G., Tao F. (2024). HacA, a key transcription factor for the unfolded protein response, is required for fungal development, aflatoxin biosynthesis and pathogenicity of *Aspergillus flavus*. Int. J. Food Microbiol..

[37] Zoupa E., Pitsikas N. (2021). The nitric oxide (NO) donor sodium nitroprusside (SNP) and its potential for the schizophrenia therapy: Lights and shadows. Molecules.

[38] Xiang F., Zhao Q., Zhao K., Pei H., Tao F. (2020). The efficacy of composite essential oils against aflatoxigenic fungus *Aspergillus flavus* in Maize. Toxins.

[39] Bonamore A., Boffi A. (2008). Flavohemoglobin: Structure and reactivity. IUBMB Life.

[40] de Jesús-Berríos M., Liu L., Nussbaum J.C., Cox G.M., Stamler J.S., Heitman J. (2003). Enzymes that counteract nitrosative stress promote fungal virulence. Curr. Biol..

[41] Turrion-Gomez J.L., Eslava A.P., Benito E.P. (2010). The flavohemoglobin BCFHG1 is the main NO detoxification system and confers protection against nitrosative conditions but is not a virulence factor in the fungal necrotroph *Botrytis cinerea*. Fungal Genet. Biol..

[42] Zhou S., Fushinobu S., Nakanishi Y., Kim S.W., Wakagi T., Shoun H. (2009). Cloning and characterization of two flavohemoglobins from *Aspergillus oryzae*. Biochem. Biophys. Res. Commun..

[43] Zhou S., Fushinobu S., Kim S.W., Nakanishi Y., Maruyama J., Kitamoto K., Wakagi T., Shoun H. (2011). Functional analysis and subcellular location of two flavohemoglobins from *Aspergillus oryzae*. Fungal Genet. Biol..

[44] te Biesebeke R., Levasseur A., Boussier A., Record E., van den Hondel C.A., Punt P.J. (2010). Phylogeny of fungal hemoglobins and expression analysis of the *Aspergillus oryzae* flavohemoglobin gene *fhbA* during hyphal growth. Fungal Biol..

[45] Zhang Z., Hao Z., Chai R., Qiu H., Wang J., Wang Y., Sun G. (2022). The flavohemoglobin gene MoFHB1 is involved in the endurance against nitrosative stress in *Magnaporthe oryzae*. FEMS Microbiol. Lett..

[46] Yang K., Luo Y., Sun T., Qiu H., Geng Q., Li Y., Liu M., Keller N.P., Song F., Tian J. (2024). Nitric oxide-mediated regulation of *Aspergillus flavus* asexual development by targeting TCA cycle and mitochondrial function. J. Hazard. Mater..

[47] Nishimura A., Kawahara N., Takagi H. (2013). The flavoprotein Tah18-dependent NO synthesis confers high-temperature stress tolerance on yeast cells. Biochem. Biophys. Res. Commun..

