# Supplementary material for: Role of Flavohemoglobins in the Development and Aflatoxin Biosynthesis of Aspergillus flavus
Source: J Fungi (Basel). 2024 Jun 19;10(6):437. doi: 10.3390/jof10060437 (PMC11204391; doi:10.3390/jof10060437)
Supplement: Supplementary file 1 [file jof-10-00437-s001.zip › jof-3032392-supplementary.pdf]

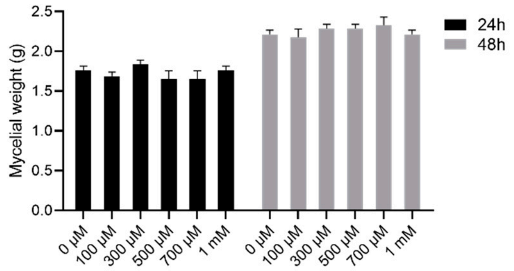

Figure S1 Effects of SNP on the growth of *A. flavus* WT strain. The strain was inoculated in YES liquid medium for 2 days, followed by treatment with varying concentrations of SNP. After further cultivation for 24 and 48 h, 20 mL of culture was sampled for each concentration, and the mycelial weight was measured.

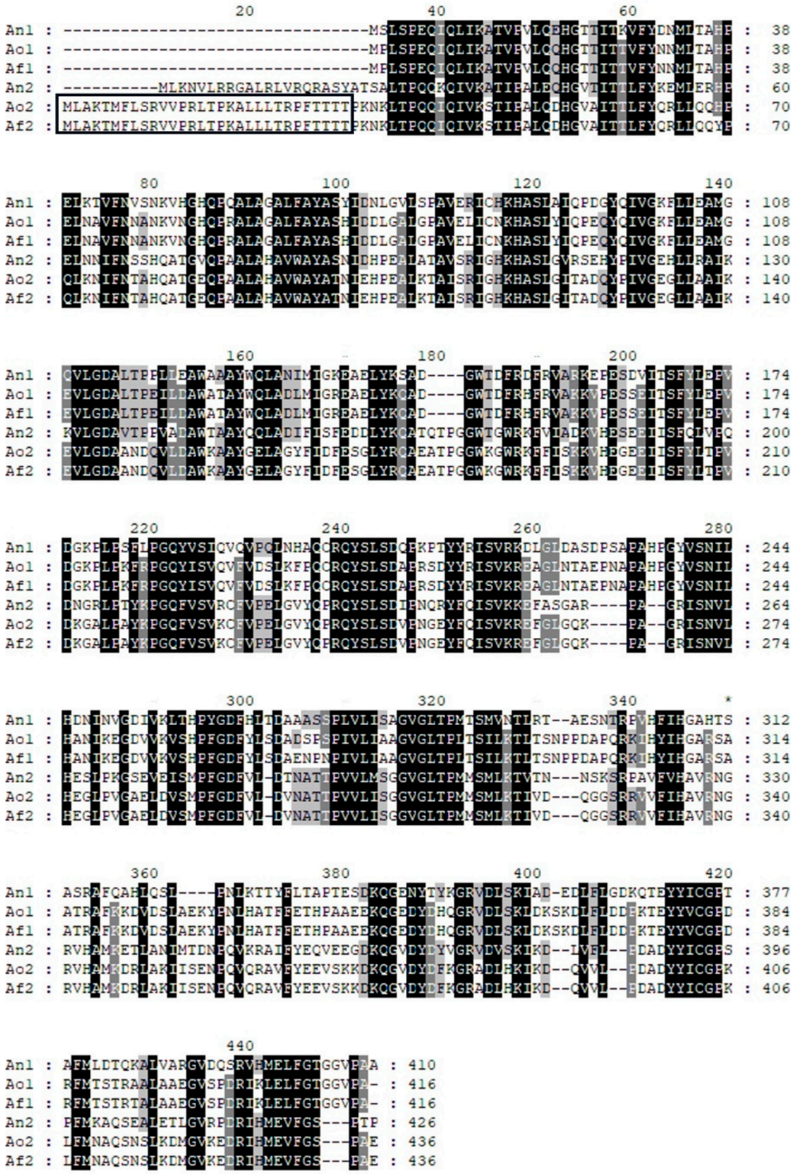

Figure S2 Primary sequence alignment of FHbA and FHbB from *A. flavus*, *A. oryzae* and *A. nidulans*. The boxed sequences represent the predicted signal peptide. Note: An1 (*fhbA*) [*A. nidulans* FGSC A4] [XP_664773.1]; An2 (*fhbB*) [*A. nidulans* FGSC A4][XP_661126.1]; Ao1(*fhb1*)[*A. oryzae*

RIB40][XP_001825874.1]; Ao2 (*fhb2*) [*A. oryzae* RIB40][XP_001727230.1]; Af1(*fhbA*)[*A. flavus* NRRL3357][AFLA_040120] ; Af2 (*fhbB*) [*A. flavus* NRRL3357] [AFLA_014530].

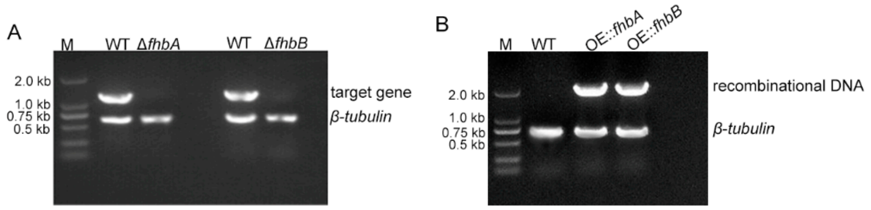

Figure S3 The genes deletion mutant and overexpression strain were validated by PCR. (A) Δ*fhbA* and Δ*fhbB* strains were identified by double PCR. Total DNA was isolated from the mycelia of *A. flavus*. The primers pairs FHbA_null_f/ FHbA_null_r and FHbB_null_f/ FHbB_null_r were used to amplify *fhbA* and *fhbB* gene, respectively. Ptub-f/ Ptub-r were used to amplify the reference gene *β-tubulin*. (B) OE::*fhbA* and OE::*fhbB* were identified by double PCR. The primer pair P100-F/ P100-R was used for positive PCR with *β-tubulin* as the reference gene.

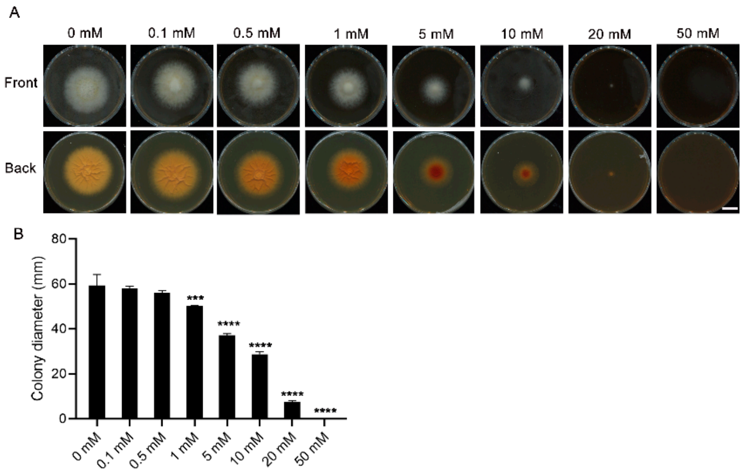

Figure S4 Effects of SNP on the growth of WT strain. (A) The morphology of colonies. WT was inoculated on YES plate supplemented with different concentrations SNP and cultured in darkness at 28 ℃ for 3 d. bar=2 cm; (B) Colony diameter. ***$p < 0.001$; ****$p < 0.0001$.

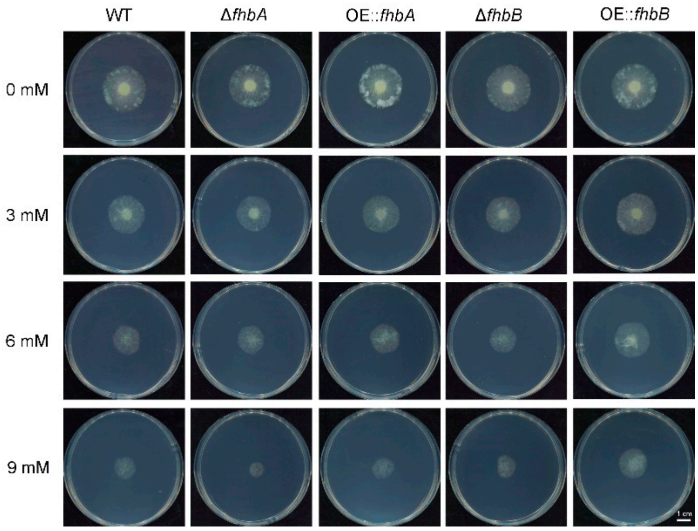

Figure S5 Colonies of the indicated strains subjected to H_2O_2. All strains were inoculated on CDA supplemented with H_2O_2 at 28 ℃ in dark for 4 d.

Table S1 Primers used in this study

Primers	Sequence: 5′to 3′	Remark
FHbA_5f	GCGGTGAGCGTATTGTAGAG	Upstream flanking region of *fhbA* gene
FHbA _5r	CCGTGGTATTTATACGGCTG	
FHbA _3f	TCCTCTCCTGCCGTTATGAGTTAC	Downstream flanking region of *fhbA* gene
FHbA _3r	TACCAGAATTACCCCTGGTTCATG	
FHbB _5f	CGTGAACCTATTGAACGACATCAG	Upstream flanking region of *fhbB* gene
FHbB _5r	CTATCCCTATGTAACCCATTTGAC	
FHbB _3f	TTGATGTACTGTTGGGGTCCTG	Downstream flanking region of *fhbB* gene
FHbB _3r	GGAGTTTCCTTCATCGCTGTTC	
Pbr-f	TGGTGTCAAGGAGGAGTAAG	Ble-RFP cassette
Pbr-r	GTCGAGTGGAGATGTGGAG	
FHbA-null-f	ATGCCGCTCTCCCCTGAACAAATC	Double pCR for Δ*fhbA* null mutant
FHbA-null-r	AGAAAACGAAACTAAGCAGGAACAC	
FHbB-null-f	AATGCTAGCCAAAACAATGTTCCTC	Double pCR for Δ*fhbB* null mutant
FHbB-null-r	TATTCTTCTGTTTATTCAGCCGGAG	
Ptub-f	AGGTCGGCAACCAGAAATATG	Double pCR for *β-tubulin* gene
Ptub-R	TTGAGTTGACCAGGGAATCG	

P101	TGGCAGGATATATTGTGGTGTAAACAAAT<br>TAGGGTATCTGTGGAAGCTGTG	*Sdh2* left flank
P102	CAGTTAAGAATGGTGAGGCAACG	
P103	CTACCGTTGCCTCACCATTC	3′ end *sdh2* gene and downstream of the *sdh2* gene
P104	CAGTTACGGAACAAAGGTCG	
S-PgPdA-f	CCGATTTTGCCGACCTTTGTTCCGT<br>AACTGTGGTGTCAAGGAGGAGTAAG	*gpdA* promoter
S-PgPdA-r	GGTGATGTCTGCTCAAGC	
OE::*fhbA*-f	AACAGCTACCCCGCTTGAGCAGACATCA<br>CCATGCCGCTCTCCCCTGAACAAATC	*fhbA* gene coding region
OE::*fhbA*-r	AGCTGTTTGATGATTTCAGTAACGTTAAG<br>TAACGAAACTAAGCAGGAACAC	
OE::*fhbB*-f	AACAGCTACCCCGCTTGAGCAGACATCA<br>CCATGCTAGCCAAAACAATGTTC	*fhbB* gene coding region
OE::*fhbB* -r	AGCTGTTTGATGATTTCAGTAACGTTAAG<br>TTATTCTTCTGTTTATTCAGCCGGAG	
S-TrpC-f	ACTTAACGTTACTGAAATCATC	*TrpC* terminator
S-TrpC-r	TCCGGCGGGCCGATCCATAACCTTCACAT<br>GTCGAGTGGAGATGTGGAGTG	
P105	CATGTGAAGGTTATGGATCG	Right flank of *sdh2*
P106	TAAACGCTCTTTTCTCTTAGGTTTACCCGC<br>TTGTCTGGGTCGGAGTTGCTCTG	
P100-F	ATGGCTGCTCTTCGCTCAACCTC	OE::*fhb*s verification
P100-R	TTGGGAGCTCGGTATAAGCTCTC	
D-FHbA-f	GGTGACTTTTACCTCTCGGAC	qRT-PCR for *fhbA* gene
D-FHbA-r	CGCCGTGGATGTAGTGAATC	
D-FHbB-f	AGTTCGGGTTGGGACAGAAG	qRT-PCR for *fhbB* gene
D-FHbB-r	GCCACCACTAATAAGCACGAC	
PtubRT-f	ATAACGAGGCCCTCTACGAC	qRT-PCR for *β-tubulin* gene
PtubRT-r	TTGAGTTGACCAGGGAATCG	
